# Contrasting impact of rural, versus urban, living on glucose metabolism and blood pressure in Uganda

**DOI:** 10.12688/wellcomeopenres.15616.2

**Published:** 2020-08-24

**Authors:** Richard E. Sanya, Irene Andia Biraro, Margaret Nampijja, Christopher Zziwa, Carol Nanyunja, Denis Nsubuga, Samuel Kiwanuka, Josephine Tumusiime, Jacent Nassuuna, Bridgious Walusimbi, Stephen Cose, Ponsiano Ocama, Richard K. Grencis, Alison M. Elliott, Emily L. Webb

**Affiliations:** 1 Immunomodulation and Vaccines Programme, Medical Research Council/ Uganda Virus Research Institute and London School of Hygiene and Tropical Medicine Uganda Research Unit, Entebbe, Uganda; 2 Department of Internal Medicine, School of Medicine, College of Health Sciences, Makerere University, Kampala, Uganda; 3 Maternal and Child Wellbeing Unit, African Population and Health Research Center, Nairobi, Kenya; 4 Department of Clinical Research, London School of Hygiene and Tropical Medicine, London, UK; 5 Faculty of Biology, Medicine and Health, University of Manchester, Manchester, UK; 6 MRC Tropical Epidemiology Group, Department of Infectious Disease Epidemiology, London School of Hygiene and Tropical Medicine, London, UK

**Keywords:** Rural, Urban, Metabolic, Hypertension, Diabetes, Insulin resistance, Helminths, Africa

## Abstract

**Background:** The burden of cardiometabolic diseases, including cardiovascular diseases and diabetes, is increasing in sub-Saharan Africa and this has been linked to urbanisation. Helminths, through their immunomodulatory properties, may protect against these disorders. We hypothesised that the rural environment protects against cardiometabolic diseases and that helminths may influence rural-urban disparity of cardiometabolic disease risk.

**Methods:** We compared metabolic parameters of individuals aged ≥10 years living in rural, high-helminth-transmission and urban, lower-helminth-transmission settings in Uganda. Cross-sectional surveys were conducted in rural Lake Victoria island fishing communities and in urban sub-wards in Entebbe municipality. Helminth infection and outcomes, including insulin resistance (computed using the homeostatic model assessment of insulin resistance [HOMA-IR]), fasting blood glucose, fasting blood lipids, blood pressure, body mass index (BMI), waist and hip circumference, were assessed.

**Results:** We analysed 1,898 rural and 930 urban participants. Adjusting for BMI, exercise, smoking, alcohol intake, age and sex, urban residents had lower mean fasting glucose (adjusted mean difference [95%CI] 0.18 [-0.32, -0.05] p=0.01) and HOMA-IR (-0.26 [-0.40, -0.11] p=0.001) but higher blood pressure (systolic, 5.45 [3.75, 7.15] p<0.001; diastolic, 1.93 [0.57, 3.29] p=0.006). Current helminth infection did not explain the observed differences.

**Conclusions:** In the Ugandan context, living in rural fishing communities may protect against hypertension but worsen glucose metabolism.

## Introduction

Globally, non-communicable diseases (NCDs) are the leading cause of mortality and disability. In 2016, they contributed to 72.3% of deaths ^1^, and cardiometabolic disorders were the top contributors to disability adjusted life years ^2^. The World Health Organisation (WHO) estimates that 78% of NCD related deaths occur in low and middle income countries and that the NCD burden is rapidly increasing in these countries ^3^. Sub-Saharan Africa, a region undergoing rapid economic growth, is experiencing this epidemiological transition, with the double burden of NCDs and communicable diseases.

The increased NCD burden has been linked to urbanisation and, currently, more than half of the world’s population lives in urban areas ^4^. Urban living is associated with traditional risk factors for cardiometabolic disease such as diets rich in energy-dense foods and reduced physical activity. This may suggest that the rural environment is protective against cardiometabolic diseases and various data sources confirm this ^5– 7^. However, there are reports of an increasing or already high burden of NCDs such as hypertension and diabetes in rural areas ^8– 10^ and no difference in burden between urban and rural settings ^11^. An increase in body mass index in rural areas has been identified as the main driver of the global epidemic of obesity ^12^.

The hygiene hypothesis proposes that the increase in chronic inflammatory disorders in high-income countries is linked to cleaner environments and less exposure to infectious agents ^13^. Exposure to infectious agents such as helminths modulates the immune system and may provide protection against these disorders. The urban environment is associated with less exposure to helminths compared to the rural environment. Recently, chronic inflammation has been linked to the aetiology of cardiometabolic disorders such as type 2 diabetes ^14^ and atherosclerosis ^15^, and has been proposed to have a role in essential hypertension ^16^. It remains unclear whether reduced exposure to helminth infections has contributed to the increase, and rural-urban differences, in cardiometabolic disease. There is evidence from animal studies, and the few published human studies, that through their effects on inflammation and metabolism, helminth infections are associated with favourable metabolic outcomes ^17– 19^.

We therefore tested the hypothesis that the rural environment is protective against metabolic risk factors and diseases and that helminths play a role in this. We investigated differences in metabolic parameters between a rural, high helminth transmission setting and an urban, lower helminth transmission setting in Uganda. We also investigated whether helminths might explain any differences observed between the two settings.

## Methods

### Study design and setting

**Figure 1.**
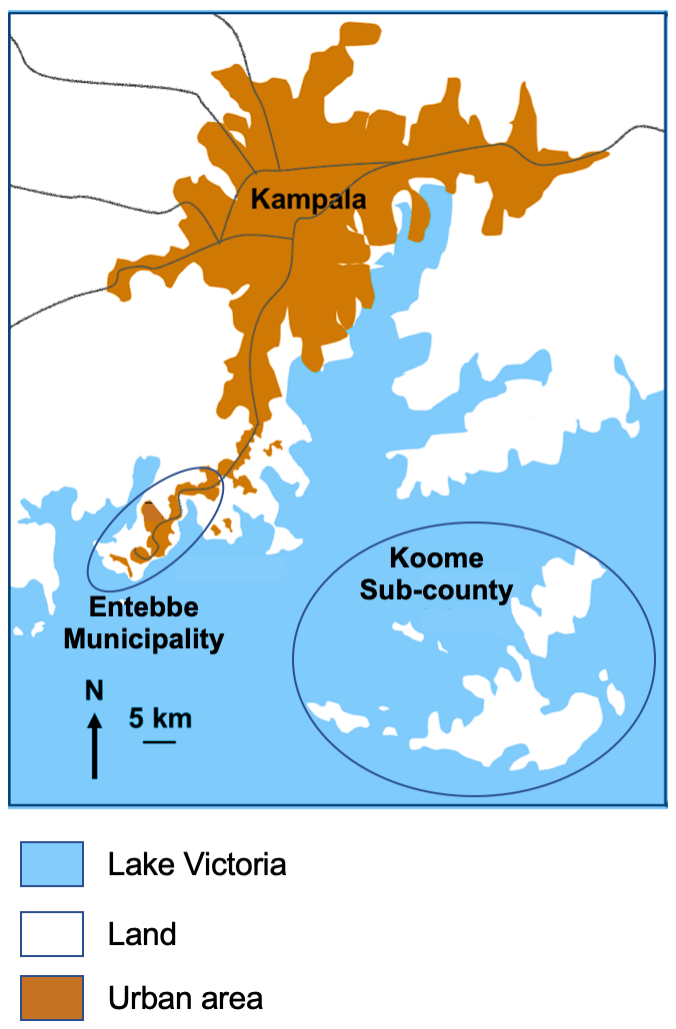
Location of the study areas.

We conducted parallel cross-sectional surveys, one in a rural and one in an urban setting ( Figure 1). The rural survey was the metabolic outcomes survey of the *Lake Victoria Island Intervention Study on Worms and Allergy-related diseases* (LaVIISWA). LaVIISWA was a cluster-randomised trial investigating the effects of intensive versus standard anthelminthic intervention on health outcomes in 26 rural Lake Victoria island communities of Koome sub-county, Mukono district, Uganda (population, 18,778) ^20, 21^. The metabolic outcomes survey was conducted between April and November 2017 after four years of the anthelminthic intervention ^22^. The survey in the urban setting was deliberately formulated to collect data in parallel with the LaVIISWA outcome surveys to enable rural-urban comparison of allergy-related ^23, 24^ and metabolic outcomes. It was conducted in Entebbe municipality, Wakiso district, Uganda from September 2016 to September 2017. Entebbe municipality is classified by the Uganda Bureau of Statistics as an urban area ^25^, and is located on the northern shores of Lake Victoria, with a population of 69,958 residing in 24 sub-wards (the smallest administrative units) ^25^.

### Participants

Household surveys were conducted in both settings. Households with members available at the time of conducting the surveys were eligible for inclusion. In the rural setting, the study team, in collaboration with the local leaders, maintained an updated register of all the households in the 26 study villages. This was used as a sampling frame to randomly select 70 households in each of the 26 study villages using Stata software (College Station, Texas, USA). In the urban setting, a different sampling technique was used because resources and time could not permit a complete household listing before starting the survey. With the help of locally available maps, we mapped each sub-ward onto satellite imagery of the area excluding areas that were uninhabited. The mapped sub-wards were then divided into segments of equal geographical size based on lines of latitude and longitude (degrees, minutes, seconds position format) and each segment was numbered. Using random number generation, segments were randomly selected from each sub-ward. The number of segments selected was proportional to the population size of the sub-ward. The midpoint of each selected segment was identified by its coordinates using a using a geographic information system (GIS) device (eTrex ^®^, Garmin™ Ltd, Kansas, United States). This was used as the starting point for sampling households and the nearest house was selected for inclusion. Houses were then sequentially selected, the next house to be sampled being the nearest to the previous house. In total, 120 geographical segments were targeted in the 24 sub-wards and in each segment, four households were targeted. If a household was empty or refused, the next one was approached until the number per segment was completed.

In both surveys, a household was defined as a habitable roofed structure whose primary function was residence or, if used for dual purposes, had at least one active resident using the structure as their primary residence. In selected and participating households, permission was sought from the household head or another adult in the household if the household head was absent. Households where all members refused to participate, or where all members were absent during the survey period, were excluded. All survey questions are described in the Codebook of the *Underlying data* ^26^.

### Variables

The exposure variables were the rural/urban setting and helminth infection status. The outcomes were insulin resistance (measured using the homeostatic model assessment of insulin resistance (HOMA-IR; HOMA-IR = fasting serum insulin x fasting glucose / 22.5), fasting blood glucose, total cholesterol, triglyceride levels, High Density Lipoprotein (HDL) - cholesterol and low density lipoprotein (LDL) – cholesterol, blood pressure, body mass index (BMI), waist and hip circumference. Following a reviewer’s suggestion, we also included mean pancreatic beta cell function (HOMA-B) as an outcome. HOMA-B was calculated using the formula, HOMA-B=(20 × fasting insulin (μIU/ml)/fasting glucose (mmol/ml) − 3.5)].

### Data sources/ study procedures

The tools and procedures of both surveys were aligned to allow comparison of the metabolic outcomes data between urban and rural settings. A questionnaire was administered to consenting household members. With this questionnaire, data were collected on household and individual sociodemographic characteristics as well as information on lifestyle, exercise or vigorous physical activity, diet, history of diabetes and hypertension.

A physical examination was performed and information obtained on blood pressure, weight, height, waist and hip circumference. With the participant seated, rested and comfortable, blood pressure was measured using a digital sphygmomanometer (OMRON Model M2[hem-7121-E], Omron Health Care, Kyoto, Japan). Three blood pressure measurements were taken five minutes apart, with the average of the last two measurements used for the analysis. A portable, flat digital weighing scale (SECA model 875 7021094, Hamburg, Germany) was used to take two measurements of body weight for each participant and the average of the two readings computed and used in the analysis. Height was measured using a stadiometer (SECA model 213 1721009, Hamburg, Germany) and recorded to the nearest millimetre. Two readings were obtained and the mean was used in the analysis. Quality control checks, using standard calibration rods for the stadiometers and standard weights for the weighing scales, were performed at the beginning of each working day. The sphygmomanometers were calibrated by the Uganda Bureau of Standards.

After an overnight fast, participants provided venous blood samples. The tests carried out on these samples included fasting blood glucose, insulin, fasting lipid profile and haemoparasitology. Fasting blood glucose, insulin and fasting lipid profile were tested using the COBAS *cobas* 6000 analyser ( *cobas* c 501 module, Roche Diagnostics, Rotkreuz, Switzerland). *Mansonella perstans* was tested using the modified Knott’s method ^27^.

Each participant was requested to provide one stool sample. Duplicate slides were made from each sample and the slides independently examined using the Kato Katz technique ^28^ by experienced technicians. Additionally, real-time stool polymerase chain reaction (PCR) was used to detect *Schistosoma mansoni*, *Necator americanus* and *Strongyloides stercoralis* ^29, 30^.

Diabetes and impaired fasting glucose were defined, according to the WHO classification, as fasting plasma glucose of ≥7.0 mmol/L and 6.1–6.9 mmol/L, respectively ^31, 32^. Hypertension was defined as diastolic blood pressure of ≥90 mmHg or systolic blood pressure of ≥140 mmHg in participants ≥18 years of age ^33^. Participants <18 years old were categorised to be hypertensive if their systolic or diastolic blood pressures were above the 95 ^th^ percentile using the Centers for Disease Control blood pressure charts for children and adolescents ^34^.

### Sample size

In the rural setting, we aimed to recruit 1950 participants (sampling 70 households without replacement was expected to yield 75 participants aged ≥10 years from each of the 26 villages). This number was primarily calculated for the LaVIISWA trial analysis and was estimated to give 80% power to detect a difference in mean HOMA-IR of 0.05 between the trial arms assuming an intra-cluster correlation coefficient of 0.03 and a standard deviation on the log scale of 0.2. In the urban survey, we targeted a sample size of 960 individuals aged ten and over, and estimated that this would allow us to detect an absolute difference of 0.03 in mean HOMA-IR between urban and rural settings, assuming a standard deviation on the log scale of 0.2, and a design effect of 1.5. ** Assuming a 13% failure rate in household response and an average of around 2.3 individuals aged ≥10 years per household 480 households were targeted.

### Statistical methods

The statistical analyses were performed using Stata version 13.0 (College Station, Texas, USA). We tabulated the characteristics of households and individuals in the urban setting alongside those of the rural setting in order to see how these differed between the two settings. We compared the outcomes between the rural setting and the urban setting. We summarised the mean for each outcome in the two settings and then assessed for differences by fitting regression models for each outcome that included a random effect to allow for the clustering (by village for the rural setting, by sub-ward for the urban setting) and a binary covariate that denoted whether the individual is in the urban or rural setting. Crude and adjusted mean differences and 95% confidence intervals are presented. All the outcomes were initially adjusted for age and sex and further adjusted for occupation, exercise, alcohol intake and smoking. HOMA-IR, fasting glucose, triglycerides, LDL-cholesterol, HDL-cholesterol, systolic and diastolic blood pressure were additionally adjusted for BMI. ** We did not perform any adjustments for multiple testing. In all analyses, data from the rural setting comprised all participants regardless of whether they had received intensive or standard anthelminthic treatment, i.e. both arms of the LaVIISWA trial were included. This was done because there were no strong differences in the outcomes between the trial arms ^22^.

To investigate whether helminths influence the differences observed in the metabolic outcomes, each helminth variable was added separately to the model and any change in the mean difference, confidence interval and p-values were assessed. Helminths were considered to have an influence on the urban-rural differences if addition of the variable resulted in a substantial change in the mean difference.

### Ethical approval and consent

Ethical approval was granted by the Uganda Virus Research Institute Research Ethics committee (reference number GC/127/17/01/573), the Uganda National Council for Science and Technology (reference number HS 2185) and the London School of Hygiene and Tropical Medicine (reference number 9917). Permission to conduct the surveys was granted by the local leadership of Mukono district (for the rural survey) and Entebbe municipality (for the urban survey). All participating adults and emancipated minors provided written informed consent. Participants aged 10 to 17 years provided assent and their parents/guardians provided written informed consent.

## Results

### Participants

**Figure 2.**
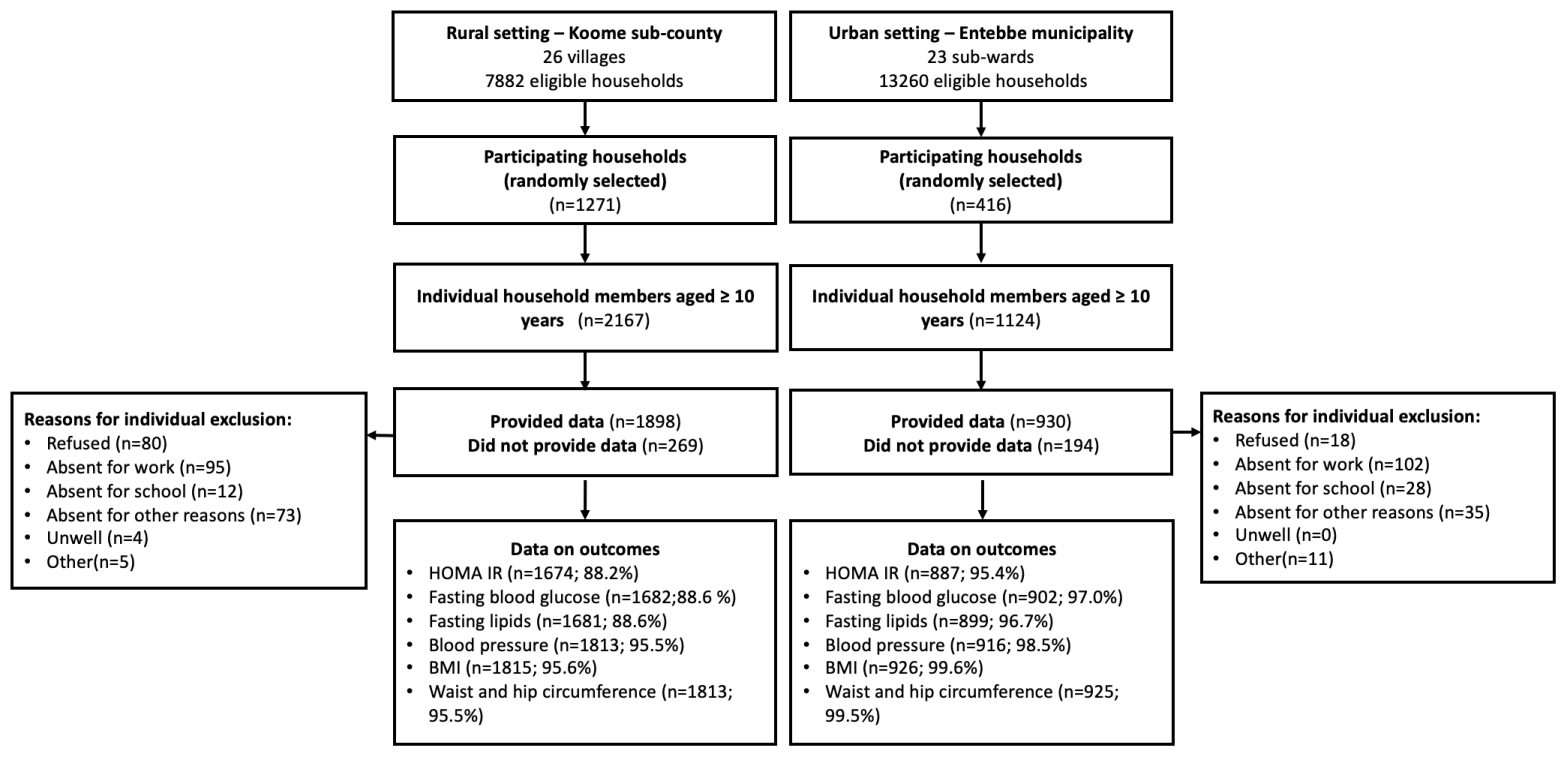
Study flow chart.

The survey flow is shown in Figure 2. In the rural setting, 2167 individuals aged 10 years and above from 1271 households were eligible for participation. Of these, 1898/2167 (87.6%) consented and provided data. In the urban setting, 1124 individuals from 416 households were eligible and 930/1124 (82.7%) consented and provided data. One sub-ward was a military facility and could not be surveyed because the study team was not granted access. In both surveys, the main reasons for non-participation were absenteeism (180/2167;8.3% in the rural setting and 165/1124;14.7% in the urban setting) and refusal (80/2167;3.7% in the rural setting and 18/1124;1.6% in the urban setting). Individual-level demographic data and survey responses are available as *Underlying data* ^26^.

### Descriptive data

The characteristics of the study participants are shown in Table 1. Male participants constituted approximately half (51%; 1010/1890) and 35% (322/920) of the participants in the rural and urban survey respectively. The mean age of the participants was 31.5 years in the rural setting and 29.7 years in the urban setting. In the rural survey, the main economic activity was fishing (739/1898;37.9%) while in the urban setting most participants were involved in service provision, were artisans or had shops and salons (211/917;23.9%). The rural population was more physically active with 49.3% (920/1868) reporting exercise or vigorous physical activity at least once a week compared to 16.6% (152/914) in the urban setting. More participants in the rural setting reported having ever smoked (343/1868;18.3% vs 38/914;4.2%) or taken alcohol (905/1868;47.5% vs 187/914;20.5%) compared to those in the urban setting.

**Table 1.** Characteristics of participants in the rural and urban settings.

Household-level characteristics	Rural setting n/N (%)	Urban setting n/N (%)	P value *
Total number of households participating in the survey	1271	416	
Household size (median, IQR)	2 (1,3)	4 (2,5)	
**Individual-level characteristics**			
Sex, male	1010/1898 (51.2)	322/920 (35.0)	<0.001
Age			
Age in years (mean, SD)	31.5 (11.0)	29.7 (15.0)	0.003
Age in years, grouped			
10–19	215/1898 (11.8)	261/920 (28.4)	<0.001
20–29	648/1898 (33.7)	298/920 (32.4)	
30–39	594/1898 (30.7)	164/920 (17.8)	
40+	441/1898 (23.7)	197/920 (21.4)	
Occupation			
Child/student	121/1898 (7.2)	253/917 (27.6)	<0.001
Housewife	199/1898 (9.7)	166/917 (18.0)	
Fishing or lake related	739/1898 (37.9)	17/917 (1.9)	
Shops, salons, artisans, service providers	214/1898 (13.0)	211/917 (23.9)	
Bars, restaurants, food providers, entertainment	160/1898 (8.4)	48/917 (5.2)	
Agriculture, lumbering, charcoal	396/1898 (19.2)	35/917 (3.8)	
Professional	33/1898 (2.3)	37/917 (4.0)	
Unemployed	36/1898 (2.4)	128/917 (14.0)	
Other (not specified)	0	22/917 (2.4)	
Residence			
Always lived in the study area	164/1898 (8.6)	454/919 (49.4)	<0.001
Place of birth			
Village	1716/1898 (90.2)	318/918 (34.6)	<0.001
Town or city	182/1898 (9.8)	600/918 (65.4)	
First five years			
Village	1700/1885 (89.8)	339/918 (36.9)	<0.001
Town or city	185/1885 (10.2)	579/918 (63.1)	
Age of participant when he/she moved to this village (mean, SD)	23.0 (11.2)	22.8 (11.3)	0.388
Parental tribe			
Maternal tribe region of origin			
Central	678/1898 (36.7)	400/918 (43.5)	0.097
Western	294/1898 (15.3)	190/918 (20.7)	
Eastern	405/1898 (21.0)	131/918 (14.3)	
Northern	202/1898 (10.1)	110/918 (12.0)	
Non-Ugandan	311/1898 (16.6)	81/918 (8.8)	
Do not know	8/1898 (0.4)	6/918 (0.7)	
Paternal tribe region of origin			
Central	751/1898 (39.1)	404/918 (44.0)	0.202
Western	328/1898 (17.6)	172/918 (18.7)	
Eastern	385/1898 (20.6)	152/918 (16.6)	
Northern	201/1898 (10.3)	115/918 (12.5)	
Non-Ugandan	230/1898 (12.2)	71/918 (7.7)	
Do not know	3/1898 (0.2)	4/918 (0.4)	
Treatment for worms			
Ever treated for worms	1661/1896 (86.9)	646/888 (72.8)	<0.001
Treated with albendazole in the last 12 months	1301/1597 (82.1)	480/641 (74.9)	0.666
Treated with praziquantel in the last 12 months	1113/1604 (67.2)	19/639 (3.0)	<0.001
Lake contact			
Frequency of lake contact			
Every day	1309/1857 (67.5)	30/916 (3.3)	<0.001
Almost every day	297/1857 (16.3)	36/916 (3.9)	
Once a week	192/1857 (11.9)	44/916 (4.8)	
Once a month	52/1857 (3.8)	120/916 (13.1)	
Less than once a month	7/1857 (0.6)	686/916 (74.9)	
Diabetes			
History of diabetes			
Yes	7/1868 (0.5)	26/914 (2.8)	<0.001
No	1841/1868 (98.7)	808/914 (88.4)	
Do not know	20/1868 (0.9)	80/914 (8.8)	
Hypertension			
History of hypertension			
Yes	44/1868 (3.0)	77/914 (8.4)	<0.001
No	1815/1868 (96.5)	811/914 (88.7)	
Do not know	9/1868 (0.5)	26/914 (2.8)	
Exercise			
How often do you exercise / participate in vigorous physical activity			
Every day	28/1868 (1.4)	11/914 (1.2)	<0.001
Almost every day	396/1868 (21.1)	53/914 (5.8)	
Once a week	496/1868 (27.3)	88/914 (9.6)	
Once a month	372/1868 (18.7)	90/914 (9.9)	
Less than once a month	576/1868 (31.5)	672/914 (73.5)	
History of smoking and alcohol intake			
Ever smoked (either pipe or cigarette)	343/1868 (18.3)	38/914 (4.2)	<0.001
Ever taken alcohol	905/1868 (47.5)	187/914 (20.5)	<0.001
Helminth infections			
*Schistosoma mansoni*, stool Kato Katz	440/1505 (31.7)	76/770 (9.9)	<0.001
*Schistosoma mansoni* intensity, stool Kato Katz			
Uninfected	1065/1505 (68.3)	694/770 (90.1)	<0.001
Light	230/1505 (16.6)	36/770 (4.7)	
Moderate	121/1505 (8.6)	27/770 (3.5)	
Heavy	89/1505 (6.5)	13/770 (1.7)	
*Schistosoma mansoni*, stool PCR	694/1487 (47.6)	171/771 (22.2)	<0.001
Hookworm, stool Kato Katz	39/1505 (2.4)	25/769 (3.3)	0.308
Hookworm, stool PCR	55/1364 (3.7)	43/771 (5.6)	0.145
*Trichuris trichiura,* stool Kato Katz	122/1505 (6.9)	17/770 (2.2)	0.002
*Ascaris lumbricoides,* stool Kato Katz	3/1505 (0.2)	0	-
*Mansonella perstans,* modified Knott’s	20/1677 (1.1)	3/918 (0.3)	0.143
*Strongyloides stercoralis,* stool PCR	101/1486 (6.1)	25/771 (3.2)	0.026

Percentages adjusted for survey design; *P values obtained from survey design-based regression.

The rural participants had more exposure to the lake with 67.5% (1309/1857) reporting daily lake contact compared to only 3.3% (30/916) in the urban setting. Regarding anthelminthic treatment, 86.9% (1661/1896) of participants in the rural setting reported having ever received treatment for worms compared to 72.8% (646/888) of urban participants. Of these, 67.2% (1113/1604) in the rural setting reported having been treated with praziquantel in the last 12 months compared to 3.0% (19/639) in the urban setting. Helminth prevalence was higher in the rural setting with a significantly higher prevalence of *S. mansoni* (stool Kato Katz, 31.7% vs 9.9%, p<0.001; stool PCR 47.6% vs 22.2%, p<0.001), *Trichuris trichiura* (stool Kato Katz, 6.9% vs 2.2%, p=0.002) and *Strongyloides stercoralis* (stool PCR, 6.1% vs 3.2%, p=0.026). In both settings, most of the *S. mansoni* infections were of light intensity.

### Rural-urban differences in metabolic outcomes

The metabolic outcomes measured in both settings are shown in Table 2. Mean fasting glucose was higher in the rural setting than in the urban setting (4.81 vs 4.71 mmol/L; adjusted mean difference -0.26 [95% confidence interval -0.40, -0.11] p=0.001). In unadjusted analyses and following adjustment for age and sex, no differences were seen in HOMA-IR. However, after further adjustment for BMI, exercise, alcohol intake and smoking, there was a negative mean difference suggesting higher HOMA-IR in the rural setting. The mean difference was most altered by adjusting for BMI. Following inclusion of HOMA-B as an additional outcome at the suggestion of a review, we found that individuals in the rural setting had lower (worse) pancreatic beta cell function than individuals in the urban setting even after adjusting for age, sex, exercise, alcohol intake, smoking and BMI (141.34 vs 201.72; 40.36 [8.00, 72.72] p=0.02].

**Table 2.** Comparison of metabolic outcomes in the urban and rural settings.

Outcome	Mean		Crude mean difference (95% CI)	P-value	*Adjusted mean difference (95% CI)	*P-value	**Adjusted mean difference (95% CI)	**P-value
	Rural	Urban						
HOMA – IR	GM 1.86	GM 2.40	0.08 (-0.07, 0.23)	0.28	-0.02 (-0.16, 0.13)	0.79	**-0.18 (-0.32, -0.05)**	**0.01**
Fasting glucose (mmol/L)	4.81	4.71	-0.10 (-0.20, 0.00)	0.05	-0.10 (-0.20, 0.01)	0.06	**-0.26 (-0.40, -0.11)**	**0.001**
Fasting insulin ( *µ*U/ml)	GM 66.99	GM 87.26	0.12 (-0.03, 0.26)	0.11	0.01 (-0.12, 0.15)	0.84	-0.12 (-0.25, 0.01)	0.06
Triglycerides (mmol/L)	1.10	1.16	0.07 (-0.05, 0.19)	0.27	0.10 (-0.03, 0.22)	0.12	0.10 (-0.03, 0.23)	0.12
Total cholesterol (mmol/L)	4.48	4.58	0.10 (-0.21, 0.40)	0.53	0.12 (-0.18, 0.41)	0.42	0.15 (-0.14, 0.44)	0.31
LDL – Cholesterol (mmol/L)	2.66	2.85	0.19 (-0.22, 0.60)	0.35	0.19 (-0.22, 0.60)	0.35	0.21 (-0.27, 0.70)	0.37
HDL – Cholesterol (mmol/L)	1.42	1.31	-0.11 (-0.35, 0.12)	0.34	-0.10 (-0.33, 0.13)	0.38	-0.07 (-0.37, 0.23)	0.63
Systolic blood pressure (mmHg)	114.21	117.28	3.07 (1.60, 4.53)	<0.001	**5.66 (4.35, 6.96)**	**<0.001**	**5.45 (3.75, 7.15)**	**<0.001**
Diastolic blood pressure (mmHg)	75.81	76.49	0.67 (-0.45, 1.80)	0.24	**2.23 (1.26, 3.21)**	**<0.001**	**1.93 (0.57, 3.29)**	**0.006**
Body mass index (kg/m2)	23.40	23.83	0.43 (-0.12, 0.97)	0.12	**0.81 (0.40, 1.23)**	**<0.001**	**1.07 (0.59, 1.54)**	**<0.001**
Waist circumference (cm)	80.44	78.95	-1.49 (-3.42, 0.43)	0.13	0.24 (-1.35, 1.82)	0.77	0.24 (-1.71, 2.19)	0.81
Waist-hip Ratio	GM 0.68	GM 0.71	0.02 (-0.01, 0.05)	0.26	0.02 (-0.01, 0.05)	0.11	0.03 (-0.01, 0.06)	0.17

HOMA-IR, Homeostatic model assessment of insulin resistance; GM, geometric means; P values obtained from survey design-based linear regression; *Adjusted for age and sex; **Adjusted for age, sex, occupation, exercise, alcohol intake and smoking; HOMA-IR, fasting glucose, triglycerides, LDL-cholesterol, HDL-cholesterol, systolic and diastolic BP were additionally adjusted for BMI.

**Figure 3.**
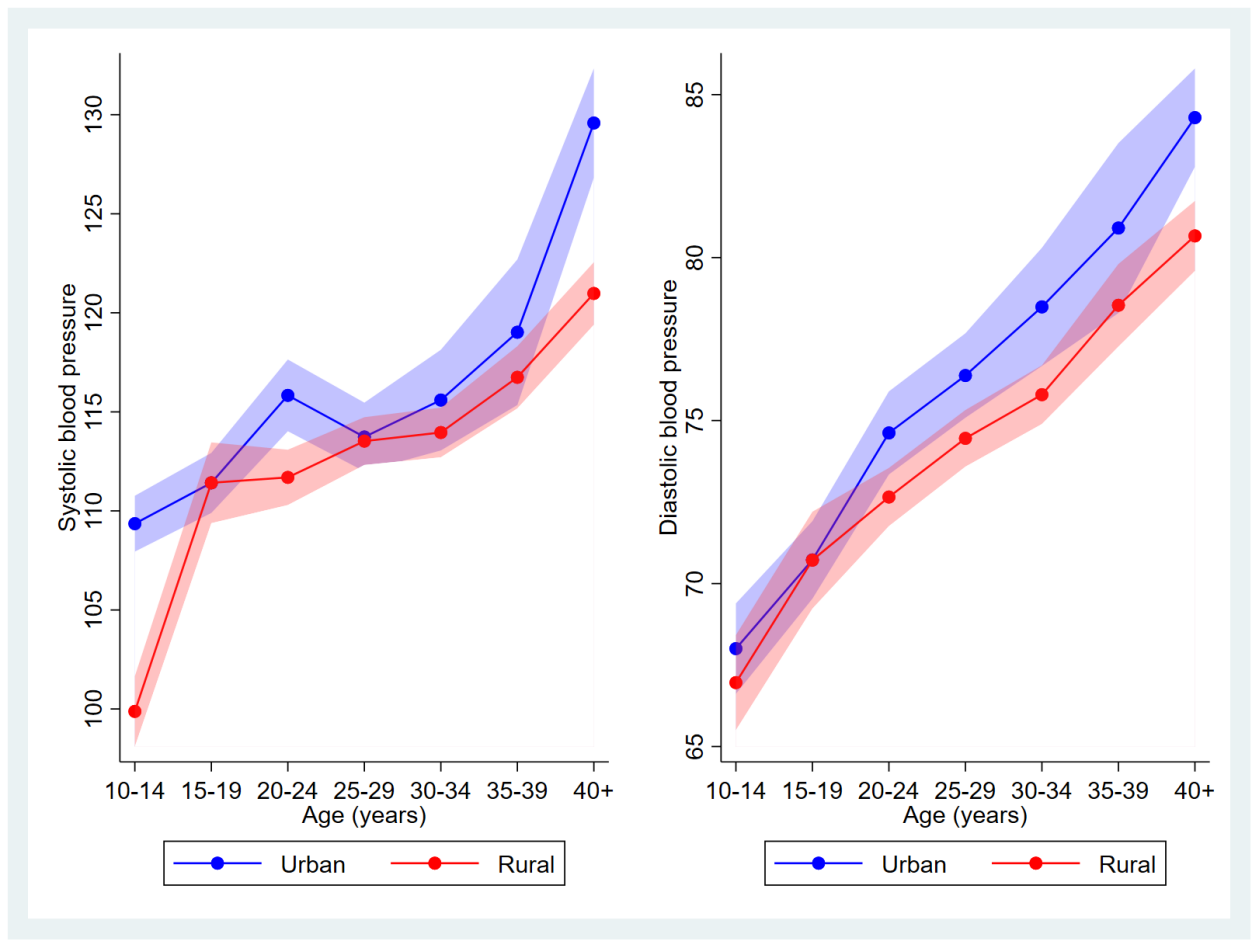
Variation of mean systolic and diastolic blood pressure with age in the rural and urban settings. The shaded areas represent 95% confidence intervals (allowing for clustering) around the mean systolic and diastolic blood pressure measurements for each age group.

Participants in the rural setting had significantly lower mean blood pressure than those in the urban setting even after adjustment for sex, age, exercise, alcohol, smoking and BMI (systolic blood pressure, 114.21 vs 117.28 mmHg 5.45 [3.75, 7.15] p<0.001; diastolic blood pressure 75.81 vs 76.49 mmHg, 1.93 [0.57, 3.29] p=0.006). These differences in blood pressure were more marked in the older age groups ( Figure 3).

Participants in the rural setting had lower mean BMI than those in the urban setting. No differences were observed for waist circumference, waist-hip ratio, triglycerides, total cholesterol, LDL-cholesterol or HDL-cholesterol.

Despite having, on average, higher fasting glucose in the rural setting, there was no difference in the prevalence of diabetes between the rural and urban settings after adjusting for multiple potential confounders. However, the rural setting had a lower prevalence of hypertension (9.1% vs 13.4%, p=0.01) and obesity (7.7% vs 14.0%, p<0.001), than the urban setting ( Table 3 and Table 4).

**Table 3.** Comparison of metabolic outcomes by disease categories.

Outcome	n/N (%)		Crude odds ratios (95% CI)	P-value	*Adjusted odds ratios (95% CI)	*P-value	**Adjusted odds ratios (95% CI)	**P-value
	Rural	Urban						
Diabetes (FBG ≥ 7 mmol/l)	16/1681 (1.0)	22/902 (2.4)	2.41 (1.32, 4.40)	0.01	**2.45 (1.31, 4.58)**	**0.01**	1.84 (0.79, 4.27)	0.15
Impaired fasting glucose (FBG 6.1 – 6.9 mmol/l)	35/1681 (1.9)	14/902 (1.6)	0.84 (0.39, 1.81)	0.66	0.92 (0.42, 2.06)	0.84	1.05 (0.42, 2.63)	0.92
Hypertension ***	156/1813 (9.1)	123/916 (13.4)	1.60 (1.14, 2.25)	0.01	**2.36 (1.67, 3.34)**	**<0.001**	**1.81 (1.15, 2.83)**	**0.01**
Prehypertension	574/1813 (30.7)	285/916 (31.1)	1.10 (0.89, 1.36)	0.36	**1.58 (1.26, 1.98)**	**<0.001**	**1.64 (1.16, 2.33)**	**0.01**
Obese (BMI ≥ 30)	128/1815 (7.7)	130/ 926 (14.0)	2.34 (1.70, 3.22)	<0.001	**2.28 (1.60, 3.23)**	**<0.001**	**2.36 (1.55, 3.60)**	**<0.001**
Overweight (BMI 25.0 – 29.9)	349/1815 (19.9)	188/ 926 (20.3)	1.30 (0.99, 1.70)	0.06	1.33 (1.00, 1.77)	0.05	1.45 (0.96, 2.17)	0.08
Underweight (BMI <18.5)	119/1815 (7.0)	132/ 926 (14.3)	2.61 (1.72, 3.96)	<0.001	**1.54 (1.04, 2.29)**	**0.03**	1.00 (0.56, 1.78)	0.99
Central (abdominal) obesity (WC≥94cm males, WC ≥80cm females)	511/1813 (29.7)	321/925 (34.7)	1.26 (0.94, 1.68)	0.12	1.15 (0.83, 1.59)	0.38	1.32 (0.86, 2.04)	0.20
Central (abdominal) obesity (WHR>0.9 males, WHR>0.85 females)	505/1813 (27.8)	227/925 (24.5)	0.84 (0.63, 1.12)	0.24	0.75 (0.55, 1.02)	0.06	0.74 (0.53, 1.03)	0.08
Metabolic Syndrome ****	109/1675 (6.4)	77/908 (8.5)	1.37 (0.88, 2.13)	0.16	1.30 (0.83, 2.04)	0.24	0.74 (0.53, 1.03)	0.08

FBG, Fasting Blood Glucose; SBP, Systolic Blood Pressure; DBP, Diastolic Blood Pressure; BMI, Body Mass Index; WC, Waist Circumference; WHR, Waist Hip Ratio; *Adjusted for age and sex; P values obtained from survey design-based logistic regression; **Adjusted for age, sex, occupation, exercise, alcohol intake and smoking; diabetes, impaired fasting glucose, hypertension and prehypertension were additionally adjusted for BMI; *** SBP ≥ 140mmHg or DBP ≥ 90mmHg for adults; Blood pressure ≥90 ^th^ percentile for participants aged 10–17 years; ****Any three of: Central obesity [WC≥94cm males WC ≥80cm Females], FBG>5.55, [SBP≥130 or DBP≥85], Elevated Triglycerides [≥1.7 mmol/L], Reduced HDL – Cholesterol [<1 mmol/L Males, <1.3 mmol/L females.

**Table 4.** Comparison of metabolic outcomes by disease categories in adult participants (aged ≥18 years).

Outcome	n/N (%)		Crude odds ratios (95% CI)	P-value	*Adjusted odds ratios (95% CI)	*P-value
	Rural	Urban				
Diabetes (FBG ≥ 7 mmol/l)	16/1551 (1.1)	21/716 (2.9)	2.66 (1.45, 4.89)	0.002	**2.34 (1.24, 4.40)**	**0.01**
Impaired fasting glucose (FBG 6.1 – 6.9 mmol/l)	33/1551 (1.9)	12/716 (1.7)	0.89 (0.40, 2.01)	0.78	0.94 (0.41, 2.13)	0.87
Hypertension **	152/1678 (9.5)	116/727 (16.0)	2.01 (1.46, 2.77)	<0.001	**2.62 (1.86, 3.69)**	**<0.001**
Obese (BMI ≥ 30)	127/1679 (8.3)	128/732 (17.5)	2.64 (1.92, 3.64)	<0.001	**2.31 (1.64, 3.26)**	**<0.001**
Overweight (BMI 25.0 – 29.9)	346/1679 (21.4)	177/732 (24.2)	1.41 (1.08, 1.84)	0.01	1.32 (1.00, 1.74)	0.05
Underweight (BMI <18.5)	51/1679 (3.2)	33/732 (4.5)	1.73 (0.96, 3.14)	0.07	1.90 (1.08, 3.36)	0.03
Central (abdominal) obesity (WC≥94cm males, WC ≥80cm females)	505/1677 (31.9)	310/731 (42.4)	1.57 (1.17, 2.13)	0.004	1.18 (0.85, 1.63)	0.32
Central (abdominal) obesity (WHR>0.9 males, WHR>0.85 females)	471/1677 (28.2)	202/731 (27.6)	0.97 (0.73, 1.29)	0.85	0.80 (0.59, 1.08)	0.14
***Metabolic Syndrome	108/1547 (6.9)	76/719 (10.6)	1.59 (1.04, 2.44)	0.03	1.30 (0.84, 2.02)	0.24

FBG, Fasting Blood Glucose; SBP, Systolic Blood Pressure; DBP, Diastolic Blood Pressure; BMI, Body Mass Index; WC, Waist Circumference; WHR, Waist Hip Ratio; *Adjusted for age and sex; P values obtained from survey design-based logistic regression; ** SBP ≥ 140mmHg or DBP ≥ 90mmHg for adults; Blood pressure ≥90 ^th^ percentile for participants aged 10–17 years; ***Any three of: Central obesity [WC≥94cm males WC ≥80cm Females], FBG>5.55, [SBP≥130 or DBP≥85], Elevated Triglycerides [≥1.7 mmol/L], Reduced HDL – Cholesterol [<1 mmol/L Males, <1.3 mmol/L females.

Helminth infection and intensity did not explain the rural urban differences observed for the metabolic parameters: very little change was seen in the mean differences after further adjustment for helminth infection status or helminth infection intensity ( Table 5).

**Table 5.** Effect of adjusting for helminths on differences in metabolic outcomes.

Outcome	Mean		Crude mean difference (95% CI)	P-value	*Adjusted mean difference (95% CI)	*P- value	**Adjusted mean difference (95% CI)	**P- value
	Rural	Urban						
**HOMA – IR**	GM 1.86	GM 2.40	0.08 (-0.07, 0.23)	0.28	-0.02 (-0.16, 0.13)	0.79	**-0.13 (-0.25, -0.01)**	**0.04**
With each helminth included in the model								
*S. mansoni* KK (n=2092)			0.07 (-0.08, 0.21)	0.38	-0.01 (-0.15, 0.14)	0.95	-0.12 (-0.25, 0.02)	0.09
*S. mansoni* PCR (n=2077)			0.05 (-0.10, 0.19)	0.54	-0.03 (-0.17, 0.11)	0.70	-0.13 (-0.25, 0 .00)	0.06
*S. mansoni* intensity KK (n=2092)			0.07 (-0.08, 0.21)	0.37	0.00 (-0.15, 0.14)	0.97	-0.11 (-0.24, 0.02)	0.09
*T. trichiura* KK (n=2092)			0.08 (-0.07, 0.22)	0.32	-0.02 (-0.16, 0.13)	0.82	-0.12 (-0.25, 0.01)	0.07
Hookworm PCR (n=1962)			0.07 (-0.08, 0.22)	0.33	-0.02 (-0.16, 0.13)	0.82	-0.12 (-0.25, 0.02)	0.08
*S. stercoralis* PCR (n=2076)			0.06 (-0.09, 0.21)	0.41	-0.02 (-0.17, 0.13)	0.78	-0.12 (-0.26, 0.01)	0.06
Any worm (n=2028)			0.05 (-0.10, 0.19)	0.54	-0.03 (-0.17, 0.11)	0.66	-0.13 (-0.25, 0.00)	0.05
**Fasting glucose (mmol/L)**	4.81	4.71	-0.10 (-0.20, 0.00)	0.05	-0.10 (-0.20, 0.01)	0.06	**-0.13 (-0.24, -0.01)**	**0.04**
With each helminth included in the model								
*S. mansoni* KK (n= 2109)			-0.10 (-0.22, 0.02)	0.09	-0.07 (-0.19, 0.04)	0.21	-0.11 (-0.23, 0.02)	0.08
*S. mansoni* PCR (n=2094)			-0.12 (-0.24, 0.00)	0.05	-0.10 (-0.21, 0.02)	0.11	-0.13 (-0.25, 0.00)	0.04
*S. mansoni* intensity KK (n=2109)			-0.10 (-0.22, 0.02)	0.10	-0.07 (-0.19, 0.04)	0.22	-0.11 (-0.23, 0.02)	0.09
*T. trichiura* KK (n=2109)			-0.08 (-0.19, 0.03)	0.14	-0.08 (-0.19, 0.03)	0.17	-0.11 (-0.23, 0.01)	0.07
Hookworm PCR (n=1979)			-0.08 (-0.19, 0.03)	0.15	-0.07 (-0.18, 0.04)	0.20	-0.11 (-0.23, 0.02)	0.09
*S. stercoralis* PCR (n=2093)			-0.08 (-0.19, 0.04)	0.194	-0.07 (-0.19, 0.04)	0.20	-0.11 (-0.23, 0.02)	0.09
Any worm (n=2045)			-0.12 (-0.25, 0.01)	0.06	-0.10 (-0.22, 0.03)	0.12	-0.13 (-0.26, 0.00)	0.05
**Systolic blood pressure (mmHg)**	114.21	117.28	3.07 (1.60, 4.53)	<0.001	5.66 (4.35, 6.96)	<0.001	4.64 (3.23, 6.06)	<0.001
With each helminth included in the model								
*S. mansoni* KK (n=2187)			3.47 (1.93, 5.01)	<0.001	5.94 (4.51, 7.38)	<0.001	4.66 (2.93, 6.39)	<0.001
*S. mansoni* PCR (n=2172)			3.45 (1.99, 4.92)	<0.001	5.82 (4.58, 7.06)	<0.001	4.67 (3.16, 6.19)	<0.001
*S. mansoni* intensity KK (n=2187)			3.47 (1.95, 5.00)	<0.001	5.94 (4.53, 7.36)	<0.001	4.65 (2.96, 6.34)	<0.001
*T. trichiura* KK (n=2187)			3.10 (1.75, 4.45)	<0.001	5.65 (4.37, 6.92)	<0.001	4.45 (2.89, 6.00)	<0.001
Hookworm PCR (n=2054)			3.42 (1.98, 4.86)	<0.001	5.88 (4.58, 7.18)	<0.001	4.64 (3.11, 6.18)	<0.001
*S. stercoralis* PCR (n=2171)			3.49 (2.10, 4.89)	<0.001	5.83 (4.55, 7.11)	<0.001	4.65 (3.13, 6.18)	<0.001
Any worm (n=2122)			3.30 (1.90, 4.70)	<0.001	5.71 (4.49, 6.92)	<0.001	4.53 (3.04, 6.03)	<0.001
**Diastolic blood pressure (mmHg)**	75.81	76.49	0.67 (-0.45, 1.80)	0.24	2.23 (1.26, 3.21)	<0.001	1.89 (0.81, 2.97)	0.001
With each helminth included in the model								
*S. mansoni* KK (n=2187)			0.34 (-0.84, 1.51)	0.567	2.17 (1.15, 3.18)	<0.001	1.71 (0.59, 2.84)	0.004
*S. mansoni* PCR (n=2172)			0.38 (-0.72, 1.48)	0.492	2.10 (1.15, 3.05)	<0.001	1.73 (0.65, 2.80)	0.002
*S. mansoni* intensity KK (n=2187)			0.32 (-0.88, 1.51)	0.596	2.15 (1.11, 3.18)	<0.001	1.67 (0.54, 2.80)	0.005
*T. trichiura* KK (n=2187)			0.35 (-0.84, 1.54)	0.554	1.97 (0.97, 2.97)	<0.001	1.55 (0.46, 2.63)	0.006
Hookworm PCR (n=2054)			0.51 (-0.66, 1.69)	0.385	2.08 (1.07, 3.09)	<0.001	1.70 (0.59, 2.80)	0.003
*S. stercoralis* PCR (n=2171)			0.57 (-0.60, 1.74)	0.330	2.08 (1.08, 3.08)	<0.001	1.71 (0.60, 2.82)	0.003
Any worm (n=2122)			0.29 (-0.79, 1.36)	0.592	2.00 (1.06, 2.94)	<0.001	1.62 (0.59, 2.65)	0.003
**Body mass index (kg/m2)**	23.40	23.83	0.43 (-0.12, 0.97)	0.12	0.81 (0.40, 1.23)	<0.001	0.60 (0.10, 1.10)	0.020
With each helminth included in the model								
*S. mansoni* KK (n=2158)			0.19 (-0.38, 0.76)	0.499	0.88 (0.43, 1.34)	<0.001	0.71 (0.11, 1.31)	0.021
*S. mansoni* PCR (n=2143)			0.18 (-0.40, 0.76)	0.535	0.80 (0.31, 1.29)	0.002	0.64 (0.01, 1.27)	0.046
*S. mansoni* intensity KK (n=2158)			0.20 (-0.38, 0.77)	0.496	0.89 (0.43, 1.35)	<0.001	0.72 (0.11, 1.32)	0.022
*T. trichiura* KK (n=2158)			0.39 (-0.17, 0.95)	0.166	0.81 (0.37, 1.25)	0.001	0.64 (0.06, 1.22)	0.032
Hookworm PCR (n=2027)			0.48 (-0.09, 1.05)	0.094	0.87 (0.41, 1.34)	<0.001	0.73 (0.14, 1.33)	0.017
*S. stercoralis* PCR (n=2142)			0.43 (-0.14, 1.00)	0.136	0.83 (0.37, 1.29)	0.001	0.67 (0.09, 1.25)	0.024
Any worm (n=2094)			0.23 (-0.35, 0.81)	0.436	0.81 (0.34, 1.28)	0.001	0.67 (0.06, 1.28)	0.033

HOMA-IR, Homeostatic model assessment of insulin resistance; GM, geometric means; P values obtained from survey design-based linear regression; *Adjusted for age and sex: **Adjusted for age, sex, exercise, alcohol intake and smoking; HOMA-IR, fasting glucose, triglycerides, LDL-cholesterol, HDL-cholesterol, systolic and diastolic BP were additionally adjusted for BMI.

## Discussion

In this paper, we have shown that individuals in a rural, high helminth transmission setting had higher mean HOMA-IR and fasting glucose, and lower pancreatic beta cell function than those in an urban low helminth transmission setting. They also had substantially lower blood pressure and BMI than their urban counterparts. This was not explained by differences in activity levels, age or sex or (for blood pressure) BMI and we did not find any impact of helminth infection on the observed rural-urban differences.

In our relatively young study population, the prevalence of diabetes was low in both settings. It was surprising to find a higher mean fasting glucose and insulin resistance and lower pancreatic beta cell function in the rural setting. Although a higher diabetes prevalence in a rural setting compared to the urban setting has been reported in a study among secondary school students in Cameroon, there was no significant difference in mean fasting glucose in that study ^35^. One possible explanation for our finding is occupational. The majority of our rural population is involved in fishing and fishing is mainly conducted at night. Night shift work has been associated with changes in the diurnal pattern of cortisol and consequently predicts increased concentrations of cortisol ^36^. Higher cortisol levels have been associated with raised plasma glucose and insulin resistance ^37, 38^. Shift work has been linked to an increased risk of diabetes ^39^, therefore, it is important to study how night time work in these rural fishing communities impacts metabolic health. Also, in view of their occupation, we cannot rule out the possibility that individuals in the rural setting were less adherent to the instructions on overnight fasting before the blood draw.

Another possible explanation is derived from the “thrifty phenotype hypothesis”, which proposes a link between poor fetal and early postnatal nutrition, and the development of T2D in adulthood ^40^. This hypothesis proposes that malnutrition in early life impedes development of the pancreas making the pancreas more susceptible to development of diabetes. Therefore, individuals who have spent their early life in rural areas (as most of the rural survey participants had done in this study), where undernutrition is more common, are more prone to glucose dysregulation.

The prevalence of hypertension was low in both settings. The rural environment was protective against hypertension, as suggested by the lower mean blood pressure and lower hypertension prevalence. This is in agreement with previous findings in Uganda and the region ^41, 42^. Taking into account the higher levels of physical activity and lower BMI among the rural participants in our study did not alter the result, neither did adjusting for smoking and alcohol intake. This implies that other protective factors associated with the rural environment, not measured in our study, such as lower sodium intake ^43^ and less pollution could be responsible for this. However, modernisation and change in household income may overturn the protective effects of the rural environment. For example, sodium intake in rural and urban Malawi is higher than the recommended amounts ^44^. Studies to investigate the trends in cardiometabolic risk factors in rural environments such as the fishing communities we studied are therefore important.

The rural-urban differences we observed in glucose metabolism, blood pressure and BMI could not be explained by the differences in current helminth prevalence. Given the existence of helminth infection in both settings, this is not surprising. The exposure to helminth infection in this particular urban setting (a peninsula, with easy access to the lake) was perhaps not low enough to eliminate helminth effects. Immunological changes induced by helminths can persist for long periods after clearance of the helminths ^45^. Lifestyle factors and other environmental exposures may be having a stronger influence on metabolic outcomes than helminths. However, investigating the role of helminths in the epidemiological transition still remains an important and interesting prospect worth pursuing. Indeed, our previous LaVIISWA trial and observational analyses suggested that schistosomiasis infection was associated with lower serum total cholesterol and LDL-cholesterol levels and that moderate to heavy S. mansoni infection was associated with lower triglycerides, LDL-cholesterol and diastolic blood pressure levels. Intensive anthelminthic treatment resulted in higher LDL-cholesterol levels, although helminths were still present in this intensively treated group, albeit with lower intensity than in residents of villages who received standard anthelminthic treatment ^22^. Further work in an area with lower helminth prevalence is required to investigate this hypothesis.

The strengths of our project include the large sample size and the uniqueness of the study settings in relation to helminth prevalence. However, we were limited by the cross-sectional nature of the study and therefore cannot make causal inferences from the results, and cannot investigate the longevity, in the context of lifestyle and epidemiological changes, of the observed differences. We performed multiple tests and did not formally correct for this, so cannot rule out chance findings, but all analyses were pre-planned and consistency between glucose and HOMA-IR and between diastolic and systolic blood pressure lend weight to the findings. Differences in socioeconomic status and diet were not assessed and it is possible that these might partially explain differences between rural and urban settings. Fishing communities are rather unique rural communities in relation to housing, lifestyle, diet and exposure to schistosomiasis, and may not be representative of all rural communities.

In conclusion, our findings raise important questions about blood glucose and the rural environment. Is the rural environment potentially detrimental to glucose metabolism? Will the source of the next epidemic of diabetes be the rural areas even when the rural environment still remains associated with a better blood pressure and anthropometric profile?

## Data availability

### Underlying data

LSHTM Data compass: Contrasting impact of rural, versus urban, living on glucose metabolism and blood pressure in Uganda. https://doi.org/10.17037/DATA.00001528 ^26^.

This project contains the following underlying data:

- Rural-urban_survey_data.csv (individual-level rural-urban survey data).
- Rural-urban_survey_data_codebook.html (codebook for Rural-urban survey data; also lists all questions asked of study participants).

Data are available under the terms of the Creative Commons Attribution 3.0 International license (CC-BY 3.0).
