# Supplementary material for: Contrasting impact of rural, versus urban, living on glucose metabolism and blood pressure in Uganda
Source: Wellcome Open Res. 2020 Aug 24;5:39. Originally published 2020 Feb 25. [Version 2] doi: 10.12688/wellcomeopenres.15616.2 (PMC7447960; doi:10.12688/wellcomeopenres.15616.2)
Supplement: Peer review history

# Peer review: Reviewer response for version 2

**DOI:** 10.21956/wellcomeopenres.17864.r40133

**Published:** 2020-08-25

**Authors:** Michael D. Gurven

**Affiliations:** 1 Department of Anthropology, University of California, Santa Barbara, Santa Barbara, CA, USA

The authors did a good job addressing my comments.

Is the work clearly and accurately presented and does it cite the current literature?

Yes

If applicable, is the statistical analysis and its interpretation appropriate?

Yes

Are all the source data underlying the results available to ensure full reproducibility?

Yes

Is the study design appropriate and is the work technically sound?

Yes

Are the conclusions drawn adequately supported by the results?

Partly

Are sufficient details of methods and analysis provided to allow replication by others?

Yes

Reviewer Expertise:

Evolutionary anthropology, indigenous health.

I confirm that I have read this submission and believe that I have an appropriate level of expertise to confirm that it is of an acceptable scientific standard.

# Peer review: Reviewer response for version 1

**DOI:** 10.21956/wellcomeopenres.17107.r38188

**Published:** 2020-04-06

**Authors:** Bruno Guigas

**Affiliations:** 1 Department of Parasitology, Leiden University Medical Center, Leiden, The Netherlands

In the present manuscript, Sanya *et al*. have conducted a cross-sectional study comparing various metabolic parameters in both rural and urban communities in Uganda for investigating the potential impact of helminth infection and living environment on cardiometabolic diseases. Altogether, after multiple adjustments, they found that individuals living in rural environment have a lower blood pressure than urban ones, as previously reported in other studies conducted in different countries, but a surprisingly higher fasting glucose levels and HOMA-IR despite lower incidence of type 2 diabetes. Helminth infection seems not to have any significant impact on all these parameters but, as acknowledged by the authors, individuals living in urban environment have a rather high rates of infection/treatment with anthelmintic drugs, suggesting that previous exposure might have already triggered/imprinted some immune response potentially beneficial for metabolic homeostasis.

 The design of the study is solid and the large sample size available, together with subtle diagnosis of helminths infection, is clearly one of the strength of such approach. The main weakness relies on the significant differences in gender and age distribution between rural and urban communities, especially for young participants (<19 year-old; 11.8% versus 28%, respectively; p<0.001). I’d have been curious to see whether the main outcomes were still present if the analyses have been systematically done only in adults.

 I have some minor comments/questions:

- I’d suggest reporting the raw data for BMI and fasting insulin levels (used to calculate HOMA-IR) in Table 1 and 2, respectively.
- To try explaining the counterintuitive higher fasting glucose levels in individuals living in rural environment, the authors suggested that early life malnutrition in such population might affect pancreas development and predispose to metabolic dysfunctions. As such, it would have been interesting to calculate HOMA-B (20 × fasting insulin (μIU/ml)/fasting glucose (mmol/ml) − 3.5), which is a widely used index for assessing pancreatic beta cell function in humans.
- The discussion around the impact of disturbances in circadian rhythms, notably of cortisol levels, on glucose homeostasis due to nocturnal fishing activities in rural communities is definitely relevant. But it also raises a question about the eventual compliance to fasting for blood collection in such conditions. Could the authors provide a bit more information about the nutritional guidelines provided to the subjects prior to blood collection (last meal, sugar beverages…)? Same thing concerning the moment of the day where the blood pressure was measured.
- I’m not sure the term “impair glucose metabolism” used in the conclusion of the abstract is the most appropriate since both fasting plasma glucose levels and HOMA-IR, although higher in individuals living in rural than urban areas, are still far from the pathological ranges.

Is the work clearly and accurately presented and does it cite the current literature?

Yes

If applicable, is the statistical analysis and its interpretation appropriate?

I cannot comment. A qualified statistician is required.

Are all the source data underlying the results available to ensure full reproducibility?

Yes

Is the study design appropriate and is the work technically sound?

Yes

Are the conclusions drawn adequately supported by the results?

Yes

Are sufficient details of methods and analysis provided to allow replication by others?

Yes

Reviewer Expertise:

Molecular pathophysiology of metabolic diseases; Immunometabolism.

I confirm that I have read this submission and believe that I have an appropriate level of expertise to confirm that it is of an acceptable scientific standard.

# Response

**Published:** 2020-08-18

**Authors:** Richard Sanya

**Affiliations:** MRC/UVRI and LSHTM Uganda Research Unit, Uganda

The authors thank the reviewer for the comments on the manuscript and respond as follows.

**Comment: **In the present manuscript, Sanya *et al*. have conducted a cross-sectional study comparing various metabolic parameters in both rural and urban communities in Uganda for investigating the potential impact of helminth infection and living environment on cardiometabolic diseases. Altogether, after multiple adjustments, they found that individuals living in rural environment have a lower blood pressure than urban ones, as previously reported in other studies conducted in different countries, but a surprisingly higher fasting glucose levels and HOMA-IR despite lower incidence of type 2 diabetes. Helminth infection seems not to have any significant impact on all these parameters but, as acknowledged by the authors, individuals living in urban environment have a rather high rates of infection/treatment with anthelmintic drugs, suggesting that previous exposure might have already triggered/imprinted some immune response potentially beneficial for metabolic homeostasis.

The design of the study is solid and the large sample size available, together with subtle diagnosis of helminths infection, is clearly one of the strength of such approach. The main weakness relies on the significant differences in gender and age distribution between rural and urban communities, especially for young participants (<19 year-old; 11.8% versus 28%, respectively; p<0.001). I’d have been curious to see whether the main outcomes were still present if the analyses have been systematically done only in adults.

**Response:** Following the reviewer’s suggestion, we did the analysis only in adults ( table 3) and still found the differences in blood pressure and BMI. However, the differences in fasting glucose and HOMA-IR were less apparent, albeit with wider confidence intervals due to the reduction in sample size.

**Comment: **I have some minor comments/questions: 

**Comment: **I’d suggest reporting the raw data for BMI and fasting insulin levels (used to calculate HOMA-IR) in Table 1 and 2, respectively.

**Response: **Data on the mean BMI in both populations are reported in table 2 and on the weight categories classified according to BMI in table 4. In the revised version of the manuscript, we have included the mean insulin levels in table 2.

**Comment: **To try explaining the counterintuitive higher fasting glucose levels in individuals living in rural environment, the authors suggested that early life malnutrition in such population might affect pancreas development and predispose to metabolic dysfunctions. As such, it would have been interesting to calculate HOMA-B (20 × fasting insulin (μIU/ml)/fasting glucose (mmol/ml) − 3.5), which is a widely used index for assessing pancreatic beta cell function in humans.

**Response:** We calculated the mean pancreatic beta cell function in the two populations using the formula above. We found that individuals in the urban setting had higher (better) pancreatic beta cell function (HOMA-B) than individuals in the rural setting even after adjusting for age, sex, exercise, alcohol intake, smoking and BMI (141.34 vs  201.72; adjusted mean difference 40.36 [95% confidence interval 8.00, 72.72] p= 0.02). This has been included in the revised manuscript.

**Comment: **The discussion around the impact of disturbances in circadian rhythms, notably of cortisol levels, on glucose homeostasis due to nocturnal fishing activities in rural communities is definitely relevant. But it also raises a question about the eventual compliance to fasting for blood collection in such conditions. Could the authors provide a bit more information about the nutritional guidelines provided to the subjects prior to blood collection (last meal, sugar beverages…)? Same thing concerning the moment of the day where the blood pressure was measured.

**Response: **The discussion has been revised to include the possibility of non-compliance to overnight fasting. However, all participants were told to fast from food and drink overnight for at least 8 hours and not to exercise or smoke before sample collection in the morning. Plain water could be consumed as much and as often as desired.

**Comment: **I’m not sure the term “impair glucose metabolism” used in the conclusion of the abstract is the most appropriate since both fasting plasma glucose levels and HOMA-IR, although higher in individuals living in rural than urban areas, are still far from the pathological ranges.

**Response: **We have revised the conclusion to reflect this.

# Peer review: Reviewer response for version 1

**DOI:** 10.21956/wellcomeopenres.17107.r38187

**Published:** 2020-04-06

**Authors:** Michael D. Gurven

**Affiliations:** 1 Department of Anthropology, University of California, Santa Barbara, Santa Barbara, CA, USA

This large-scale cross-sectional study compares urban and rural Ugandans in several measures of cardiometabolic health risk. It highlights one salient aspect of many rural environments - intestinal helminthic infections - to test whether having helminths is associated with better cardiometabolic health. This follows from a small but growing literature that has proposed the intriguing hypothesis that helminths might offer protection against insulin resistance, hypercholesterolemia, hypertension and other factors underlying diabetes and atherosclerosis risk. While the authors find urban-rural differences in the majority of health outcomes, they do not find any evidence that helminths mediate these differences.

 As the authors indicate, helminth infection is highly prevalent in both rural and urban settings, and so there is no helminth-free control comparison. While helminths do not explain any urban-rural differences in cardiometabolic health, it is possible that helminth infection is still associated with lower blood glucose, lower blood pressure and other health variables – just that these effects are similar across the urban-rural divide. Those analyses are not reported here. But a prior paper by this same research team working in Uganda (Sanya *et al*. 2019 ^1^) does a better job of directly testing the helminth hypothesis by comparing those who received a standard antihelminth treatment with those receiving a more intensive pharmacological treatment regime. While that paper also did not have a helminth-free control, it did show that more frequent antihelminth treatment resulted in lower LDL and total cholesterol. Having a heavier *Schistosoma mansoni* infectious burden was also associated with lower blood triglyceride levels and lower diastolic blood pressure. It is curious that these rich findings by the same team are not highlighted or contextualized in the current study.

 I wonder if the Kato Katz analysis of infection intensity gives any more traction than the binary presence/absence used in the paper. Given most people have low-level burden, is it the case that protective effects are more evident among those with heavier burden? Given the cross-sectional nature of the database, however, those with heavier worm burden may have other health deficits or be living under more resource-poor and compromised setting. Additional controls for socioeconomic status may help adjust for potential confounding.

 As for the urban-rural health differences, the authors show that urban areas have higher diabetes risk (but lower fasting blood glucose), and higher prevalence of hypertension and obesity – though it is worth noting that overall prevalence of these conditions is low in both settings, and that ~77% of their sample consists of people under age 40. Perhaps due to the small absolute differences between setting, they find no urban-rural differences in metabolic syndrome—itself just a combination of the other cardiometabolic biomarkers.

 The emphasis on urban-rural health differences is a useful starting point, but itself does not explain much, and may not generalize to other regions or countries. The authors conclude in the Abstract: “In low-income countries, rural living may protect against hypertension but impair glucose metabolism”. What is the basis for making such a broad generalization? “Urban” or “rural” are proxies for a cluster of traits that directly impact health, including diet, lifestyle differences (e.g. alcohol, smoking), physical activity, infection, social cohesion, pollution exposure, and the like. In the Ugandan context here, there are some patterns that do not generalize to many other urban-rural comparisons. For example, smoking and alcohol consumption appear to be more common in the rural than the urban context. Underweight is more prevalent in the urban setting, and this urban setting has lower but non-trivial prevalence of infection by several helminth species. These characteristics will vary widely across regions and countries. In order to generalize you would expect to find consistent differences across the key domains relevant to cardiometabolic disease: diet, physical activity, lifestyle, infection, etc.

 Given that urban-rural health differences still exist in their fully adjusted models, what can one conclude as to why? The first possibility is what might be missing from the adjusted models. The most obvious missing variable(s) reflect diet. It was a shame that diet was not assessed (or even described in a general way), including additives like sugar, salt and oil, and consumption of processed foods and beverages. Physical activity was also not measured directly, though participants were asked about “exercise and vigorous activity”. Exercise may be construed as a bit of a luxury, and vigorous activity is indicative but may not be the most relevant for comparison. What is more telling is how much moderate activity vs. sedentary activity people engage in through their daily activities – not restricted to leisure or exercise. Even without activity directly measured (e.g. with accelerometry), it’s possible that occupation might give a better indication of habitual activity than the interview question on exercise/vigorous activity. Perhaps adjustment for livelihood would contribute towards explaining (at least statistically) the urban-rural health differences.

 Another question is how long the current differences between urban and rural contexts have existed? If some lifestyle changes are recent, there might be greater health differences across urban-rural contexts among the younger cohorts. In this case, there would be an interaction between age cohort and urban-rural difference. Similarly, is there a sex difference in exposures due to how modernization manifests? I noticed that the urban sample was much more female-biased, suggesting male out-migration or wage labor leading to greater male absenteeism.

 The authors mention the thrifty phenotype as a possible explanation for why people in the rural areas might be prone to glucose dysregulation. Thrifty phenotype and developmental origins of health and disease (DOHaD) paradigms emphasize the effects of early life exposures on later life chronic disease risk, especially when conditions shift over the life course (e.g. from harsh to abundant). That one-third of those living in the urban context were born and spent their first five years of life in a rural area permits one to explore the potential effects of developmental plasticity. A “purer” urban-rural contrast would be limited to those who spent their entire lives in the same environmental context. An additional exploration could test whether those who spent their early life in a rural context but now live in an urban context show worse cardiometabolic health than those who have spent their entire lives in a rural context. The authors have the data to make these comparisons, but do not include these early life variables in any analyses other than sample description (Table 1).

 Lastly, while the authors speculate that sleep disruption and altered circadian rhythms might explain the surprising finding that fasting glucose is higher in the rural context, a simpler (but less interesting) possibility is that people in a rural context may be less likely to adhere to the requirement of an overnight fast prior to blood draw (especially if as noted, night fishing is a common activity in the rural setting). In the future, glycosylated hemoglobin (HbA1c) could be measured to provide a more reliable, long-term indicator of glucose levels.

 Also, although maybe less relevant, it was unclear whether there are ethnic differences within or between sampling areas that might reflect genetic differences underlying health risk.

 Overall, this is a nice epidemiological study highlighting imminent chronic disease risks in Uganda, showcasing that such risks are not restricted to urban areas. Prevalence rates of hypertension and central obesity are noteworthy in both urban and rural contexts.

 Minor:

 Given lower response rate due to absenteeism in the urban sample, are absent individuals more or less likely to show any health differences than those at home (e.g. more involved in wage labor, and perhaps more integrated into market economy)? If data exist for only one round of data collection then maybe this potential for bias cannot be checked.

 Any current use of medications, e.g. for hypertension? If so, are those individuals excluded from analyses of biomarkers?

Is the work clearly and accurately presented and does it cite the current literature?

Yes

If applicable, is the statistical analysis and its interpretation appropriate?

Yes

Are all the source data underlying the results available to ensure full reproducibility?

Yes

Is the study design appropriate and is the work technically sound?

Yes

Are the conclusions drawn adequately supported by the results?

Partly

Are sufficient details of methods and analysis provided to allow replication by others?

Yes

Reviewer Expertise:

Evolutionary anthropology, indigenous health.

I confirm that I have read this submission and believe that I have an appropriate level of expertise to confirm that it is of an acceptable scientific standard, however I have significant reservations, as outlined above.

## References

[1] : The effect of helminth infections and their treatment on metabolic outcomes: results of a cluster-randomised trial. *Clin Infect Dis*.2019; 10.1093/cid/ciz859 10.1093/cid/ciz859 31504336PMC7384320

# Response

**Published:** 2020-08-18

**Authors:** Richard Sanya

**Affiliations:** MRC/UVRI and LSHTM Uganda Research Unit, Uganda

The authors thank the reviewer for the comments on the manuscript and respond as follows.

**Comment: **This large-scale cross-sectional study compares urban and rural Ugandans in several measures of cardiometabolic health risk. It highlights one salient aspect of many rural environments - intestinal helminthic infections - to test whether having helminths is associated with better cardiometabolic health. This follows from a small but growing literature that has proposed the intriguing hypothesis that helminths might offer protection against insulin resistance, hypercholesterolemia, hypertension and other factors underlying diabetes and atherosclerosis risk. While the authors find urban-rural differences in the majority of health outcomes, they do not find any evidence that helminths mediate these differences.

As the authors indicate, helminth infection is highly prevalent in both rural and urban settings, and so there is no helminth-free control comparison. While helminths do not explain any urban-rural differences in cardiometabolic health, it is possible that helminth infection is still associated with lower blood glucose, lower blood pressure and other health variables – just that these effects are similar across the urban-rural divide. Those analyses are not reported here. But a prior paper by this same research team working in Uganda (Sanya *et al*. 2019 ^1^) does a better job of directly testing the helminth hypothesis by comparing those who received a standard antihelminth treatment with those receiving a more intensive pharmacological treatment regime. While that paper also did not have a helminth-free control, it did show that more frequent antihelminth treatment resulted in lower LDL and total cholesterol. Having a heavier *Schistosoma mansoni* infectious burden was also associated with lower blood triglyceride levels and lower diastolic blood pressure. It is curious that these rich findings by the same team are not highlighted or contextualized in the current study.

**Response: **Thank you for this suggestion. We agree that helminth infection may still be associated with lower blood glucose and other health variables. Following the reviewer’s suggestion, in the new version of the manuscript, we have incorporated our previous findings into the fifth paragraph of the discussion, and highlight that the potential role of helminths in the epidemiological transition merits further investigation.

**Comment: **I wonder if the Kato Katz analysis of infection intensity gives any more traction than the binary presence/absence used in the paper. Given most people have low-level burden, is it the case that protective effects are more evident among those with heavier burden? Given the cross-sectional nature of the database, however, those with heavier worm burden may have other health deficits or be living under more resource-poor and compromised setting. Additional controls for socioeconomic status may help adjust for potential confounding.

**Response: **Thank you for this suggestion. We have redone the analysis and included *S. mansoni* infection intensity in the revised manuscript (Table 5). *S. mansoni* infection intensity did not explain  the rural-urban differences, and we have expanded the text in the last paragraph of the results section to include this finding. Unfortunately, we did not collect data on socio-economic status in the metabolic survey conducted in the rural setting and were therefore unable to adjust for it in this analysis. We now state this in the discussion (paragraph 6) as a limitation of the study.

**Comment: **As for the urban-rural health differences, the authors show that urban areas have higher diabetes risk (but lower fasting blood glucose), and higher prevalence of hypertension and obesity – though it is worth noting that overall prevalence of these conditions is low in both settings, and that ~77% of their sample consists of people under age 40. Perhaps due to the small absolute differences between setting, they find no urban-rural differences in metabolic syndrome—itself just a combination of the other cardiometabolic biomarkers.

**Response**: Thank you for pointing this out. The overall prevalence of diabetes and hypertension was low in both settings and the majority of the population was young. We have now highlighted the relatively young study population (representative of the country as a whole) in the discussion. We had previously stated in the discussion that the prevalence of diabetes was low in both settings, but have now included a similar statement about hypertension in the revised manuscript.

**Comment: **The emphasis on urban-rural health differences is a useful starting point, but itself does not explain much, and may not generalize to other regions or countries. The authors conclude in the Abstract: “In low-income countries, rural living may protect against hypertension but impair glucose metabolism”. What is the basis for making such a broad generalization? “Urban” or “rural” are proxies for a cluster of traits that directly impact health, including diet, lifestyle differences (e.g. alcohol, smoking), physical activity, infection, social cohesion, pollution exposure, and the like. In the Ugandan context here, there are some patterns that do not generalize to many other urban-rural comparisons. For example, smoking and alcohol consumption appear to be more common in the rural than the urban context. Underweight is more prevalent in the urban setting, and this urban setting has lower but non-trivial prevalence of infection by several helminth species. These characteristics will vary widely across regions and countries. In order to generalize you would expect to find consistent differences across the key domains relevant to cardiometabolic disease: diet, physical activity, lifestyle, infection, etc.

**Response: **We appreciate the reviewer’s point and have refined the conclusion to make it more specific. It now reads “In the Ugandan context, living in rural fishing communities may protect against hypertension but worsen glucose metabolism”.

**Comment: **Given that urban-rural health differences still exist in their fully adjusted models, what can one conclude as to why? The first possibility is what might be missing from the adjusted models. The most obvious missing variable(s) reflect diet. It was a shame that diet was not assessed (or even described in a general way), including additives like sugar, salt and oil, and consumption of processed foods and beverages. Physical activity was also not measured directly, though participants were asked about “exercise and vigorous activity”. Exercise may be construed as a bit of a luxury, and vigorous activity is indicative but may not be the most relevant for comparison. What is more telling is how much moderate activity vs. sedentary activity people engage in through their daily activities – not restricted to leisure or exercise. Even without activity directly measured (e.g. with accelerometry), it’s possible that occupation might give a better indication of habitual activity than the interview question on exercise/vigorous activity. Perhaps adjustment for livelihood would contribute towards explaining (at least statistically) the urban-rural health differences.

**Response: **In the second last paragraph of the discussion, we acknowledged the absence of data on diet as a limitation and have stated, in the revised manuscript, that the differences in the rural and urban settings may be partially explained by diet and socio-economic status, and that not being able to adjust for these factors is a limitation of the study. Following the reviewer’s suggestion, we have redone the analysis and added occupation as a possible proxy for activity levels to the adjusted models. No major differences were observed between the findings from the adjusted model and those in the previous model without the occupation variable. The results of the adjusted model are presented in the new version of the manuscript. 

**Comment: **Another question is how long the current differences between urban and rural contexts have existed? If some lifestyle changes are recent, there might be greater health differences across urban-rural contexts among the younger cohorts. In this case, there would be an interaction between age cohort and urban-rural difference. Similarly, is there a sex difference in exposures due to how modernization manifests? I noticed that the urban sample was much more female-biased, suggesting male out-migration or wage labor leading to greater male absenteeism.

**Response: **Thank you for this. In view of the cross-sectional nature of the study design, we were unable to determine how long the current differences in metabolic parameters between the rural and urban contexts have existed and to our knowledge, no study has previously assessed the urban-rural differences in metabolic outcomes in these populations. The rural population in particular is highly mobile, with only 9% having lived there all their lives (Table 1), therefore examining for an interaction between age cohort and urban-rural differences may not be informative.  We have expanded our discussion to highlight these consequences of the cross-sectional study design.

**Comment: **The authors mention the thrifty phenotype as a possible explanation for why people in the rural areas might be prone to glucose dysregulation. Thrifty phenotype and developmental origins of health and disease (DOHaD) paradigms emphasize the effects of early life exposures on later life chronic disease risk, especially when conditions shift over the life course (e.g. from harsh to abundant). That one-third of those living in the urban context were born and spent their first five years of life in a rural area permits one to explore the potential effects of developmental plasticity. A “purer” urban-rural contrast would be limited to those who spent their entire lives in the same environmental context. An additional exploration could test whether those who spent their early life in a rural context but now live in an urban context show worse cardiometabolic health than those who have spent their entire lives in a rural context. The authors have the data to make these comparisons, but do not include these early life variables in any analyses other than sample description (Table 1).

**Response: **Exploring the potential effects of plasticity on the metabolic outcomes and the “purer” rural-urban contrast limited to those who spent their entire lives in the same environmental context are excellent suggestions. However, these analyses were not preplanned and the surveys were not powered to measure them. With that in mind, we have performed this posthoc analysis and the results are shown in tables 1 and 2. We did not find differences in HOMA-IR and fasting glucose between individuals in the urban environment who spent their entire lives in the urban environment and those who were born and lived in the rural environment for the first five years and later moved to the urban environment ( table 1).

For the “purer” rural-urban analysis limited to individuals who spent their entire lives in the same environment ( table 2), we still found differences in fasting glucose and blood pressure, as in the main analysis. However, the difference in HOMA-IR, observed in the main analysis was lost here.

The number of individuals analysed was far smaller than in the main analysis, and this cannot be ruled out as a reason for the discrepancy in the two findings. However, we do appreciate the input and will factor this in when designing rural-urban comparison studies in the future.

**Comment: **Lastly, while the authors speculate that sleep disruption and altered circadian rhythms might explain the surprising finding that fasting glucose is higher in the rural context, a simpler (but less interesting) possibility is that people in a rural context may be less likely to adhere to the requirement of an overnight fast prior to blood draw (especially if as noted, night fishing is a common activity in the rural setting). In the future, glycosylated hemoglobin (HbA1c) could be measured to provide a more reliable, long-term indicator of glucose levels.

**Response:** Thank you for this suggestion - we have revised the manuscript to include this possibility.

**Comment: **Also, although maybe less relevant, it was unclear whether there are ethnic differences within or between sampling areas that might reflect genetic differences underlying health risk.

**Response: **There were no ethnic differences between the sampling areas. This is shown in table 1 where reference is made to parental tribe.

**Comment: **Overall, this is a nice epidemiological study highlighting imminent chronic disease risks in Uganda, showcasing that such risks are not restricted to urban areas. Prevalence rates of hypertension and central obesity are noteworthy in both urban and rural contexts.

Minor:

**Comment: **Given lower response rate due to absenteeism in the urban sample, are absent individuals more or less likely to show any health differences than those at home (e.g. more involved in wage labor, and perhaps more integrated into market economy)? If data exist for only one round of data collection then maybe this potential for bias cannot be checked.

**Response: **Unfortunately, there was only one urban survey. We did not explore the health differences between individuals who were present at home and those absent because the individual questionnaires used to capture these data were only administered to participants who were present.

**Comment: **Any current use of medications, e.g. for hypertension? If so, are those individuals excluded from analyses of biomarkers?

**Response:** Current use of medications especially for hypertension, was uncommon and therefore these individuals were not excluded from the analysis.
